# Patient-specific and organ-centric approach in malperfusion in acute type A dissection

**DOI:** 10.1016/j.xjon.2024.04.002

**Published:** 2024-04-09

**Authors:** Bashisth Mishra, Simiyu R. Namungu, Abdifatah A. Mohamed

**Affiliations:** a Kenyatta University Teaching, Referral & Research Hospital, Nairobi, Kenya; b London Polyclinic Parkland, Nairobi, Kenya

To the Editor:

**Figure:**
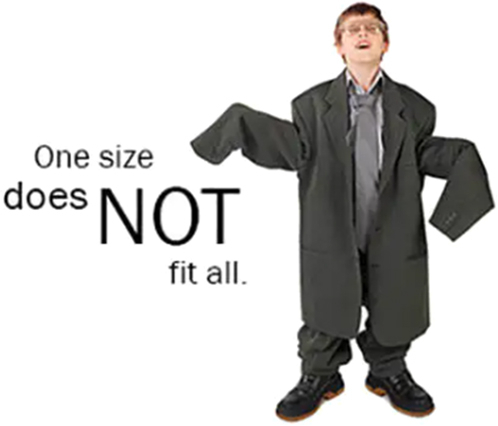

We read with interest the article by Brown and colleagues^1^ and congratulate the authors for this elegant study. In it, they recommend aortic surgery first for the treatment of acute type A aortic dissection (ATAAD) in malperfusion syndrome (MPS). MPS indicates end-organ ischemia with dysfunction, necrosis, and organ failure.

We know the strategy to address MPS in ATAAD is unsettled. The outcome of treatment for MPS is dependent on the specific organ involved and the timing of treatment for MPS.^2^ Intestinal ischemia has the greatest morbidity and mortality, and the outcomes for other organs malperfusion vary.^2^ We believe during early days of surgery for ATAAD, interventional procedures were in their infancy; hence, many of the earlier studies did not have the opportunity to use them; however, recent studies are using it more frequently.^2^^,^^3^ The most important requirement in any strategy is saving the life of a patient. We believe this complex set of patients must be first categorized as stable or unstable, as the outcomes will be different. This has been very well illustrated by Yang and colleagues^4^ in their recent article. Although debatable, the possibility of aortic rupture leading to death of the patient is less as compared with organ failure in ATAAD with MPS^3^^,^^4^! The authors^4^ of the study hypothesized that all patients with MPS would die but not all untreated patients with TAAD would have aortic rupture! The risk of dying from end-organ failure even after the branch arterial obstruction was resolved with fenestration/stenting was ≈7 times greater than the risk of aortic rupture.^4^ Sometimes it may prevent the futile attempt of aortic surgery in a patient whose visceral organ is damaged beyond salvaging.

In a clinically stable patient, the intervention to correct the ischemia in MPS should be undertaken, as it will have an effect on outcome.^2^, ^3^, ^4^ Other organs must be addressed on their own merits, keeping in mind their significance and the condition of the patient.

It is known that cerebral and renal malperfusion respond well to aortic surgery^2^ in most cases unless otherwise indicated^5^; in each clinical situation, hence, upfront aortic surgery should be the aim in these cases. Coronary malperfusion needs to be addressed before or during the operation, depending on whether myocardial ischemia or complications of acute dissection are the dominant cause of instability of the patient. In a stable patient, aortic surgery first may still be a better strategy. Limb ischemia in malperfusion needs to be addressed on its own merits, depending on severity and its clinical significance in the given context.

In conclusion, we believe the treatment of MPS in the setting of ATAAD should be individualized and specific organ involvement should be a consideration while selecting the better strategy.

## Conflict of Interest Statement

The authors reported no conflicts of interest.

The *Journal* policy requires editors and reviewers to disclose conflicts of interest and to decline handling or reviewing manuscripts for which they may have a conflict of interest. The editors and reviewers of this article have no conflicts of interest.
